# Cyproheptadine enhances weight gain and modulates appetite-regulating peptides in children with failure to thrive and food allergies

**DOI:** 10.3389/fphar.2026.1776222

**Published:** 2026-06-15

**Authors:** Yuxin Zhang, Ni Ma, Jiaoli Lan, Xiaoyan Zhang, Zhiling Li, Qing Zhao, Cuiyuan Jin, Chunfu Li, Min Yang

**Affiliations:** 1 Department of Pediatrics, Guangdong Provincial People’s Hospital (Guangdong Academy of Medical Sciences), Southern Medical University, Guangzhou, China; 2 Department of Pediatrics, Nanfang Hospital, Southern Medical University, Guangzhou, China; 3 Shenzhen Maternity and Child Healthcare Hospital, Women and Children’s Medical Center, Southern Medical University, Shenzhen, Guangdong, China; 4 Institute of Translational Medicine, Zhejiang Shuren University, Hangzhou, China

**Keywords:** appetite regulation, children, cyproheptadine hydrochloride, failure to thrive, food allergies, Nesfatin-1, NUCB2

## Abstract

**Background:**

Cyproheptadine hydrochloride (CH) possesses both antihistaminic and appetite-stimulating properties, suggesting therapeutic potential for children with failure to thrive (FTT) and food allergies. This study evaluated the effects of CH on growth parameters, clinical manifestations, and serum levels of appetite-regulating peptides Nesfatin-1 and NUCB2 in children aged 1–3 years with FTT and food allergy.

**Methods:**

In this non-randomized controlled real-world study, participants were assigned to either the treatment group (T-group, n = 25), receiving CH at 0.15 mg/kg twice daily for 12 weeks, or the control group (NT-group, n = 19). Primary outcomes were anthropometric indices: body weight, weight-for-age Z-score (WAZ), height, weight-for-length Z-score (WLZ), body mass index (BMI), and length-for-age Z-score (LAZ). Secondary outcomes included clinical symptom scores and changes in Nesfatin-1 and NUCB2 levels.

**Results:**

After 12 weeks, the T-group showed significantly greater improvements in body weight (10.32 ± 0.28 kg vs. 9.05 ± 0.23 kg, *P* = 0.002), WAZ (*P* < 0.001), WLZ (*P* < 0.001), and BMI (*P* = 0.001) compared to the NT-group. No significant differences were observed in height or LAZ. Within the T-group, significant improvements from baseline to week 12 were observed for all weight-based indices (*P* < 0.001). CH treatment was also associated with significant reductions in serum Nesfatin-1 (*P* = 0.000) and NUCB2 (*P* = 0.043) levels. At baseline, reduced food intake and sleep disturbances were negatively correlated with WAZ and LAZ, respectively.

**Conclusion:**

CH treatment significantly enhances weight gain and improves key anthropometric indices in young children with FTT and food allergy, potentially mediated by the downregulation of anorexigenic peptides Nesfatin-1 and NUCB2. These findings suggest that CH may be a promising pharmacotherapeutic option for this complex patient population.

## Introduction

Failure to thrive (FTT) is a common pediatric condition characterized by inadequate growth and weight gain, often resulting from a complex interplay of medical, nutritional, and psychosocial factors (Homan, 2016). Among the medical etiologies, food allergies have been increasingly recognized as a significant contributor to FTT, primarily due to malabsorption, inflammation, and dietary restrictions (Smith et al., 2023). The resulting malnutrition poses substantial risks for long-term developmental delays and compromised health outcomes, underscoring the need for effective therapeutic strategies that address both the allergic and nutritional components of this condition.

Cyproheptadine hydrochloride (CH), a first-generation antihistamine with additional anti-serotonergic activity, has long been used for its appetite-stimulating effects (Kapur et al., 1997). Its broad receptor affinity, particularly the antagonism of 5-HT2 receptors, underlies both its antihistaminic and orexigenic properties (Kim et al., 2021). The US Food and Drug Administration (FDA)-approved labeling for CH includes mild, uncomplicated urticaria and angioedema, and the drug is commonly used in clinical practice for allergic dermatologic conditions, including atopic dermatitis. Supporting evidence for its efficacy in chronic urticaria dates back to a 1977 study in the Archives of Dermatology (Wanderer et al., 1977). Additionally, its potential utility in infants and young children with low appetite and poor growth has been suggested (Sant’Anna et al., 2014). Several studies have documented the efficacy of CH in promoting weight gain in various pediatric populations, including undernourished children (Najib et al., 2014), those with feeding difficulties (Sant’Anna et al., 2014), and children with non-organic FTT (Lin et al., 2021). A systematic review further consolidated evidence supporting its role as an appetite stimulant (Harrison et al., 2019).

Despite this evidence, the specific utility of CH in children with the dual diagnosis of FTT and food allergy remains underexplored. This is a notable gap, given that CH’s dual pharmacologic profile—alleviating allergic symptoms while simultaneously stimulating appetite—theoretically makes it an ideal candidate for this complex patient group. It is important to note that not all children with FTT present with reduced food intake as the predominant mechanism. In cases primarily driven by malabsorption, chronic inflammation, or metabolic abnormalities, the utility of an appetite stimulant such as CH may be limited. Therefore, the use of CH should be considered specifically in children with FTT and food allergy who exhibit clinically significant anorexia or feeding aversion, as was the case in our study population.

This real-world study aimed to evaluate the clinical efficacy of CH in young children with FTT and food allergy, focusing not only on traditional growth and symptom measures but also on exploring potential mechanisms through the lens of appetite-regulating peptides Nesfatin-1 and NUCB2. We hypothesized that CH treatment would lead to significant improvements in growth parameters and that these improvements would be associated with modulation of key anorexigenic pathways.

## Methods

### Study design and participants

This was a non-randomized, controlled, real-world study conducted at the Guangdong Provincial People’s Hospital. Randomization was not performed because, given the pragmatic clinical setting and parental preferences, many families of children with significant FTT were unwilling to accept the possibility of allocation to a non-treatment arm. The assignment was therefore based on informed parental choice after detailed clinical consultation. Children aged 1–3 years diagnosed with both FTT and food allergy were eligible for enrollment. FTT was defined as a weight-for-age Z-score (WAZ) less than −2 SD below the World Health Organization (WHO) growth standard median (Homan, 2016). The diagnosis of food allergy was confirmed based on established criteria (Luyt et al., 2014; Homan, 2016): a documented history of anaphylaxis (Smith et al., 2023); clinical symptoms consistent with food allergy plus a serum-specific immunoglobulin E (sIgE) level >3.5 kUA/L with symptomatic improvement after 4 weeks of dietary elimination; or (Kapur et al., 1997) a positive oral food challenge (OFC).

Exclusion criteria included active infectious diseases, congenital genetic or metabolic disorders, primary immunodeficiencies, inflammatory bowel disease (excluded by clinical history, endoscopic findings when available, and biomarkers such as fecal calprotectin and CRP), malignancy, or major surgery within the preceding 3 months.

Based on parental preference and clinical consultation, participants were allocated to either the treatment group (T-group), which received CH syrup, or the non-treatment control group (NT-group). All children received standardized nutritional counseling, which included guidance on tolerable solid foods prepared by parents, strict avoidance of known allergenic foods, and daily supplementation with 300–400 mL of a high-energy-density amino acid-based formula (AAF, 1 kcal/mL; Neocate Junior, SHS International Ltd., Liverpool, UK). The T-group received oral CH at a dose of 0.15 mg/kg twice daily for 12 weeks, consistent with Chinese pediatric guidelines (Subspecialty Group of Gastroenterology, the Society of Pediatrics et al., 2022). The study was approved by the Medical Ethics Committee of Guangdong Provincial People’s Hospital (Ethics No: KY-Q-2022-001-01) and registered with the Chinese Clinical Trial Registry (ChiCTR2300077432). Written informed consent was obtained from all parents or legal guardians.

### Outcome measures

Primary outcomes were anthropometric growth indices, measured at baseline (week 0) and study endpoint (week 12). These included body weight (kg), height/length (cm), WAZ, length-for-age Z-score (LAZ), weight-for-length Z-score (WLZ), and BMI.

Secondary outcomes included: (Homan, 2016) Clinical symptom scores: Assessed using a predefined scoring system adapted from previous literature (McDonald et al., 2013), which evaluated vomiting, diarrhea, abdominal pain, eczema/atopic dermatitis, reduced food intake, and sleep disorders (see Supplementary Table S1 for detailed criteria). For each symptom, a score of 0 indicated absence, 1 indicated mild or moderate, and 2 indicated severe manifestations. The total score ranged from 0 to 12, with higher scores reflecting more severe symptom burden (Supplementary Table S1). Reduced food intake was defined as a decrease in oral intake by at least one-third compared to the child’s usual baseline intake, as reported by parents or primary caregivers, and scored according to Supplementary Table S1 (score ≥1). Caloric intake was not systematically quantified due to the variability in home-prepared solid foods. Total and individual symptom scores were recorded at weeks 0 and 12 (Smith et al., 2023). Serum Nesfatin-1 and NUCB2 levels: Fasting blood samples were collected at baseline and week 12. Serum levels of Nesfatin-1 and NUCB2 were quantified using commercially available enzyme-linked immunosorbent assay (ELISA) kits according to the manufacturers’ instructions. Serum levels of Nesfatin-1 and NUCB2 were quantified using commercially available ELISA kits (Nesfatin-1: Cusabio, Wuhan, China; NUCB2: MyBioSource, San Diego, CA, United States) according to the manufacturers’ instructions. The intra- and inter-assay coefficients of variation and detection limits for each kit were within the ranges reported by the manufacturers.

### Safety assessment

All adverse events (AEs), such as drowsiness, irritability, behavioral changes, or other potential side effects, were actively monitored and recorded throughout the study period. The severity, duration, outcome, and potential relationship to the study drug were assessed for each AE.

### Statistical analysis

Data are presented as mean ± standard error of the mean (SEM). Between-group comparisons of continuous variables were performed using independent samples t-tests, while categorical variables were compared using the Chi-square test or Fisher’s exact test as appropriate. The Mann-Whitney U test was used to compare total clinical symptom scores between groups. Spearman’s rank correlation coefficient was employed to assess the relationships between clinical symptom scores and growth indices. A two-tailed *P*-value of < 0.05 was considered statistically significant. All analyses were conducted using SPSS Statistics version 26.0 (IBM Corp., Armonk, NY, United States).

## Results

### Baseline demographic and clinical characteristics

A total of 44 children (mean age 19.14 ± 6.26 months; 40.91% female) were enrolled, with 25 in the T-group and 19 in the NT-group. The baseline demographic and clinical characteristics are summarized in Table 1. There were no significant differences between the two groups in terms of sex distribution, age, recent antibiotic use, proportion with positive food sIgE, diagnosis of cow’s milk protein allergy (CMPA), polysensitization (≥3 foods), or reliance on amino acid-based or extensively hydrolyzed formula. This confirms that the groups were well-matched at baseline.

**TABLE 1:** Baseline clinical characteristics of the study participants.

Characteristic	T-group (n = 25)	NT-group (n = 19)	*P* value
Female (%)	10 (40.00%)	8 (42.10%)	0.573
Age (months) (mean ± SEM)	20.44 ± 1.40	18.53 ± 1.73	0.390
Antibiotic treatment 1 month ago (%)	6 (24.00%)	8 (42.10%)	0.202
Food allergen sIgE (+) (%)	16 (64.00%)	11 (57.89%)	0.680
Diagnosed of cow`s milk protein allergy (%)	10 (40.00%)	5 (26.32%)	0.407
Allergic foods ≥3 (%)	2 (8.00%)	3 (15.79%)	0.638
Feeding with AAF/eHF (%)	22 (88.00%)	15 (79.00%)	0.443

### Effects of CH on growth indices

As shown in Table 2, no significant differences existed in any growth parameter between the T-group and NT-group at baseline. After intervention, the T-group exhibited statistically significant and clinically relevant improvements in weight, WAZ, and WLZ compared to the NT-group at week 4 and week 12. The BMI was significant differences at week 12. Specifically, at week 4 the mean weight in the T-group increased to 9.73 ± 1.31 kg versus8.76 ± 1.06 kg in the NT-group (*P* = 0.012), at week12 the mean weight in the T-group increased to 10.32 ± 0.28 kg versus 9.05 ± 0.23 kg in the NT-group (*P* = 0.002). In contrast, no significant between-group differences were observed for height or LAZ after 12 weeks.

**TABLE 2:** Comparison of growth indicators between the two groups at baseline, week 4 and week 12.

Growth indicator	Baseline (0 weeks)			Week 4			Week 12		
	T-group (n = 25)	NT-group (n = 19)	*P* value	T-group (n = 25)	NT-group (n = 19)	*P* value	T-group (n = 25)	NT-group (n = 19)	*P* value
Weight (kg)	8.54 ± 0.24	8.01 ± 0.26	0.137	9.73 ± 1.31	8.76 ± 1.06	0.012	10.32 ± 0.28	9.05 ± 0.23	0.002
Height (cm)	78.48 ± 1.09	76.08 ± 1.28	0.161	79.71 ± 5.56	77.70 ± 5.28	0.231	82.27 ± 1.10	80.66 ± 1.20	0.332
WAZ	−2.38 ± 0.07	−2.52 ± 0.12	0.299	−1.42 ± 0.70	−1.96 ± 0.66	0.012	−1.15 ± 0.12	−2.05 ± 0.18	0.000
LAZ	−1.79 ± 0.21	−2.02 ± 0.25	0.482	−1.72 ± 1.06	−1.76 ± 0.92	0.899	−1.45 ± 0.22	−1.62 ± 0.29	0.646
WLZ	−2.05 ± 0.19	−2.07 ± 0.16	0.946	−0.77 ± 1.05	−1.47 ± 0.85	0.024	−0.58 ± 0.20	−1.72 ± 0.17	0.000
BMI	13.91 ± 0.23	13.98 ± 0.19	0.842	15.38 ± 1.54	14.61 ± 1.02	0.065	15.4 ± 0.27	14.07 ± 0.21	0.001

These within-group changes confirm the growth-promoting effect of CH over time. Although birth weight was available from medical records, the primary analysis focused on anthropometric changes during the 12-week intervention period. Visual inspection of individual growth trajectories suggested a clear inflection point in weight gain after CH initiation in the T-group, but formal curve modeling was beyond the scope of this study.

### Effects on clinical symptom scores

The most common clinical manifestations at baseline were vomiting, diarrhea, abdominal pain, eczema/atopic dermatitis, reduced food intake and sleep disorders. Sleep disorder was defined as easily waking with irritability or sudden waking with crying/inconsolability, as detailed in Supplementary Table S1. No significant differences in individual or total scores were observed between the two groups at baseline or at week 12. Notably, T-group and NT-group showed significant reductions in diarrhea, reduced food intake, and sleep disorder scores from baseline to week 12, whereas the T-group showed significant improvement in eczema or atopic dermatitis, as shown in Table 3. Symptoms of vomiting or diarrhea and eczema or atopic dermatitis resolved completely (score of 0) in the T-group, while mild symptoms persisted in a few children in the NT-group.

**TABLE 3:** Clinical symptom scores of T-group and NT-group at baseline and Week 12.

Symptom	T-group (n = 25)					NT-group (n = 19)				
	0 W		12 W		*P* value	0 W		12 W		*P* value
	Score	Mean rank	Score	Mean rank		Score	Mean rank	Score	Mean rank	
Vomiting	0.12 ± 0.33	27.00	0.00 ± 0.00	24.00	0.077	0.26 ± 0.65	20.11	0.11 ± 0.32	18.89	0.567
Diarrhea	0.72 ± 0.79	32.00	0.00 ± 0.00	19.00	0.000	0.58 ± 0.69	23.55	0.05 ± 0.23	15.45	0.003
Abdominal pain	0.20 ± 0.41	26.40	0.16 ± 0.47	24.60.35	0.493	0.37 ± 0.60	21.55	0.11 ± 0.32	17.45	0.108
Eczema/atopic dermatitis	0.48 ± 0.77	29.50	0.00 ± 0.00	21.50	0.002	0.47 ± 0.61	22.08	0.16 ± 0.375	16.92	0.070
Reduced food intake	0.92 ± 0.70	30.98	0.36 ± 0.84	20.02	0.003	0.95 ± 0.78	24.18	0.26 ± 0.56	14.82	0.004
Sleep disorder	0.96 ± 0.84	32.16	0.16 ± 0.47	18.84	0.000	0.84 ± 0.77	24.71	0.11 ± 0.32	14.29	0.001
Total scores	3.44 ± 1.85	35.54	0.84 ± 1.57	15.46	0.000	3.26 ± 1.97	26.92	0.47 ± 0.91	12.08	0.000

### Correlation between clinical symptoms and growth indices

At baseline, Spearman correlation analysis in Table 4, revealed a significant negative correlation between reduced food intake and WAZ (r = −0.374, *P* = 0.012), and between sleep disorders and LAZ (r = −0.326, *P* = 0.031). No other significant linear correlations were found between other individual symptoms or the total score and the primary growth indices (WAZ and LAZ).

**TABLE 4:** Linear correlation analysis between clinical symptom scores and WAZ or LAZ at 0 week.

Symptom (n = 44)	WAZ		LAZ	
	r	*P* value	r	*P* value
Vomiting	0.012	0.939	−0.083	0.593
Diarrhoea	0.083	0.592	0.101	0.513
Abdominal pain	−0.150	0.330	−0.140	0.365
Eczema or atopic dermatitis	−0.218	0.154	0.022	0.886
Reduced food intake	−0.374	0.012	−0.102	0.509
Sleep disorder	−0.200	0.193	−0.326	0.031
Total scores	−0.258	0.090	−0.177	0.252

### Serum Nesfatin-1 and NUCB2 levels

The changes in serum levels of the appetite-regulating peptides are summarized in Table 5. At baseline, Nesfatin-1 and NUCB2 levels were comparable between the two groups. After 12 weeks, the T-group demonstrated a significant reduction from baseline in both Nesfatin-1 (*P* = 0.000) and NUCB2 (*P* = 0.000) levels. The NT-group also showed a significant reduction in NUCB2 (*P* = 0.017), but not in Nesfatin-1 (*P* = 0.386). At week 12, the NUCB2 level in the T-group was significantly lower than that in the NT-group (*P* = 0.043). The between-group difference in Nesfatin-1 levels at week 12 approached but did not reach statistical significance (*P* = 0.050).

**TABLE 5:** Serum concentrations of Nesfatin-1 and NUCB2 at baseline and week 12.

Appetite-regulating peptide	​	T-group (n = 25)	NT-group (n = 19)	*P* value
Nesfatin-1 (pg/mL)	0 weeks	1,328.47 ± 89.79	1,275.02 ± 145.34	0.159
	12 weeks	1,135.23 ± 112.7	1,225.26 ± 170.13	0.050
	*P* value	0.000	0.386	​
NUCB2(ng/mL)	0 weeks	14.20 ± 3.25	13.34 ± 2.01	0.322
	12 weeks	10.55 ± 1.65	11.61 ± 1.58	0.043
	*P* value	0.000	0.017	​

### Safety

CH treatment was well-tolerated. Only one child (4%) in the T-group experienced mild and transient drowsiness during the initial days of treatment at a dose of 0.3 mg/kg/day, which resolved spontaneously without intervention within 3 days. No other adverse events were reported in either group throughout the study period.

## Discussion

This real-world study provides evidence suggesting that CH serves as an effective pharmacotherapeutic adjunct for promoting weight gain in young children with the complex comorbidity of FTT and food allergies. Our findings demonstrate that a 12-week course of CH significantly improved key weight-based anthropometric indices—including body weight, WAZ, WLZ, and BMI—compared to dietary management alone. Both between-group comparisons and within-group longitudinal analyses demonstrated significant improvements in weight, WAZ, WLZ, and BMI in the T-group after 12 weeks of CH treatment, supporting the conclusion that CH contributed to these growth changes. The improvement of weight may be more significant in the first 4 weeks. Furthermore, we present novel mechanistic data suggesting that this growth-promoting effect may be mediated, at least in part, through the downregulation of the anorexigenic peptides Nesfatin-1 and NUCB2.

The significant anthropometric improvements observed in our CH-treated group align with and extend the existing literature on CH’s efficacy as an appetite stimulant in various pediatric populations (Sant’Anna et al., 2014; Lin et al., 2021; Harrison et al., 2019). The novelty of our work lies in its specific focus on children with concomitant FTT and food allergy, a group that stands to benefit uniquely from CH’s dual pharmacologic profile. By antagonizing histamine H1 and serotonin 5-HT2 receptors, CH may simultaneously mitigate allergic inflammation (Kacar et al., 2021) and directly stimulate central appetite pathways, thereby addressing two core pathophysiological drivers of growth failure in this population.

A central and innovative finding of our study is the significant reduction in serum levels of Nesfatin-1 and NUCB2 following CH treatment. Nesfatin-1, a potent anorexigenic peptide derived from its precursor NUCB2, is known to inhibit food intake and reduce body weight (Schalla and Stengel, 2018). Elevated levels of Nesfatin-1 have been previously reported in malnourished children (Sezer et al., 2018). The downregulation of these peptides post-CH treatment offers a plausible biochemical correlate of the observed increase in appetite and weight gain. However, whether this reflects a direct central effect of CH on hypothalamic pathways or a secondary response to improved nutritional status remains to be determined in future mechanistic studies. The more pronounced reduction of NUCB2 in the CH group compared to the control group strengthens the argument for a drug-specific effect beyond what is achieved by dietary intervention alone.

Although T-group and NT-group showed significant reductions in diarrhea, reduced food intake, and sleep disorder scores from baseline to week 12, whereas the T-group showed significant improvement in eczema or atopic dermatitis. The between-group difference in clinical symptom scores did not reach statistical significance. This is likely due to the overall low symptom burden at baseline (mean total score <3.5 in both groups) and the potent effect of dietary elimination, which also improved symptoms in the NT-group, creating a floor effect that limited further discrimination. Therefore, while CH may provide additional symptomatic benefit, the present data do not allow definitive conclusions regarding superior symptom control over dietary management alone.

The study has several limitations inherent to its real-world, non-randomized design. The non-randomized design, while ethically pragmatic, introduces potential selection bias, which we have acknowledged as a major limitation. Birth weight data were not incorporated into the growth curve analysis, which may have provided additional context for baseline growth status. The assignment based on parental preference, while ethical and pragmatic, introduces the potential for selection bias. The relatively small sample size restricts the generalize ability of the findings and limits the power for more sophisticated subgroup analyses. The 12-week duration, though sufficient to demonstrate weight gain, is too short to assess the long-term impact on linear growth (LAZ), safety, or the sustainability of the effect. Finally, the reliance on subjective symptom scores, though based on a structured tool, introduces a potential for reporter bias.

Despite these limitations, our findings have significant clinical implications. CH presents a viable, safe, and effective pharmacological option to augment dietary management in a complex patient group where growth recovery is often challenging. The modulation of Nesfatin-1 and NUCB2 provides initial mechanistic insights and positions these peptides as potential biomarkers for monitoring treatment response in future studies.

## Conclusion

This study suggests that cyproheptadine hydrochloride may serve as a useful therapeutic adjunct for enhancing weight gain in young children with failure to thrive and food allergies. The observed downregulation of Nesfatin-1 and NUCB2 provides a potential mechanistic correlate of its appetite-stimulating effects. Future randomized, placebo-controlled trials with longer follow-up needed to confirm these findings and establish the long-term efficacy and safety.

## Data Availability

The original contributions presented in the study are included in the article/Supplementary Material, further inquiries can be directed to the corresponding authors.
