# Supplementary material for: Cyproheptadine enhances weight gain and modulates appetite-regulating peptides in children with failure to thrive and food allergies
Source: Front Pharmacol. 2026 Jun 15;17:1776222. doi: 10.3389/fphar.2026.1776222 (PMC13311060; doi:10.3389/fphar.2026.1776222)
Supplement: Supplementary file 1 [file Table1.pdf]

Supplementary Table S1. Clinical Symptom Scoring Criteria

Score items	Assigned point		
	0	1	2
Vomiting	None	1-3 times/day	≥ 4 times/day
Diarrhoea	None	3 ~ 4 times/day， loose stool	≥ 5 times/day， watery or bloody stool
Abdominal pain	None	occasionally， 1-2 times/week	Intermittently， ≥3 times/week<br>With abdominal distention and satiety
Eczema or atopic dermatitis	None	1-2 places	≥ 3 places
Reduced food intake	None	Oral intake decreased by 1/3	Oral intake decreased by 2/3
Sleep disturbed	None	Easily wake-up with minimal irritability; spontaneously back to sleep	Sudden wake-up with crying; inconsolable and back to sleep
